# Exploring virulence factors, virulome, and multidrug resistance of *Klebsiella pneumoniae* strains isolated from patients with central Line-associated bloodstream infections

**DOI:** 10.1038/s41598-025-07493-6

**Published:** 2025-06-20

**Authors:** Mohab G. Sayed, Moselhy S. Mansy, Mervat I. El Borhamy, Heba M. Elsherif

**Affiliations:** 1 Department of Microbiology and Immunology, Faculty of Pharmacy, Misr International University (MIU), P.O. 19648, Cairo, Egypt; 2 Department of Microbiology and Immunology, Faculty of Pharmacy, AL-Azhar University, P.O. 11651, Cairo, Egypt; 3 Clinical Microbiology Laboratory, International Medical Center, P.O 11451, Cairo, Egypt

**Keywords:** *Klebsiella pneumoniae*, CLABSI, Extended-spectrum beta-lactamase, Multidrug resistance, Microbiology, Diseases, Medical research

## Abstract

This study aimed to analyze the antibiotic resistance patterns and virulence profiles of *Klebsiella pneumoniae*, a prevalent gram-negative pathogen linked to CLABSI patients globally. Of a total of 185 microbial isolates, 51 (27.5%) were *K*. *pneumoniae* isolates. The results of antimicrobial susceptibility testing using the disk diffusion method were compared to those of the VITEK-2. Phenotypic analysis revealed that 88.3% were biofilm producers and 50.9% were extended-spectrum beta-lactamase (ESBL) producers. Enterobacterial repetitive intergenic consensus-polymerase chain reaction (ERIC-PCR) revealed a discriminatory power of 0.7111 between ten selected isolates. The PCR detection of the virulence genes, including *FimH*, *rmpA*, *iutA*, and *fyuA*, revealed that the ten selected isolates harbored all these genes, except one without the *fyuA* gene. The presence of the *rmpA* and the *iutA* genes confirmed them as hypervirulent (hv) *K*. *pneumoniae*. The genes (*EAST-1*, *CNF-1*) were present in 20% and 60% of the isolates, respectively. All isolates had the *bla*_*TEM*_ and *bla*_*SHV*_ resistance genes, while 50% had the *bla*_*NDM*_ carbapenemase resistance gene. In conclusion, all selected *K*. *pneumoniae* isolates were proven to be ESBL producers and carbapenem-resistant, highlighting significant multidrug resistance. We found a strong correlation between biofilm formation and multidrug resistance, as well as between phenotypic and genotypic detection of various virulence factors. Infections from hyKp strains represent a growing public threat. Our findings aim to enhance therapeutic options for patients and help reduce complications and mortality.

**Supplementary Information:**

The online version contains supplementary material available at 10.1038/s41598-025-07493-6.

## Introduction

Central line-associated bloodstream infection (CLABSI) is among the most serious health-associated infections (HAIs). It has been associated with increased hospitalization, healthcare costs, and mortality, especially in developing countries^1–3^. Several pathogens may be probable causes of CLABSIs, but the primary pathogens of greatest concern are *Klebsiella pneumoniae*, followed by Staphylococcus spp., *Acinetobacter baumannii*, and *Pseudomonas aeruginosa*^4^. The increased occurrence of multidrug-resistant (MDR-GNB) bacteria, with the dramatic increase in the prevalence of extended-spectrum beta-lactamase (ESBL) producing *K. pneumoniae* and *E. coli*, as well as carbapenem-resistant Enterobacteriaceae (CRE), has become a serious challenge for healthcare professionals due to the limited treatment options available for patients^5^.

The diversity of clinical infections caused by *K. pneumoniae* is linked to various virulence factors, such as capsules, adhesions, biofilm formation, and iron acquisition systems. Capsule production is crucial for immune evasion during initial infection and persistence in the bloodstream. Adherence, mediated by fimbriae and other adhesins, may be essential in the earlier phases of bacteremia when pathogens bind to specific receptors at the initial infection site and disseminate to the blood. Biofilms promote the persistence of microorganisms and facilitate bacterial dissemination^6,7^. A new variant of *K. pneumoniae* called hypervirulent *K. pneumoniae* (hvKp) has been reported worldwide, causing more serious community and hospital-acquired infections. It can be distinguished from classical *K. pneumoniae* based on its hypermucoviscosity^8^. Therefore, this study focused on the phenotypic detection of several virulence profiles of *K. pneumoniae*, including biofilm formation, ESBL production, hypermucoviscosity, and multidrug resistance, along with the virulome analysis, in comparison with *E. coli* and *A. baumannii*, with emphasis on hypervirulent strains isolated from patients with CLABSIs.

## Methods

### Clinical specimens

Unidentified blood specimens were collected from the clinical microbiology laboratory of a tertiary care hospital from patients admitted to different ICUs from May 2019 to March 2022. The selection criteria included those with primary bacteremia who exhibited fever before reaching its peak, as well as patients presenting with hypotension and an elevated heart rate. The research was conducted as per the Ethical Code 04-Egypt-Code-of-Medical-Ethics-Ministry-of-Health-and-Population-238/2003-part four. This study was conducted following the ethical principles of the Declaration of Helsinki. The study was approved by the institutional ethical committee of the Faculty of Pharmacy, Ain Shams University (ACUC-FP-ASU RHDIRB2020110301).

### Identification and antimicrobial susceptibility testing

The identification of gram-negative isolates was performed according to Bergy’s Manual using standard identification procedures. Antibiotic susceptibility was analyzed by the disc diffusion method as recommended by the Clinical and Laboratory Standards Institute 2020 (CLSI)^9^. The concentrations of the antibiotic discs used (expressed in µg) were as follows: amikacin (30), ampicillin (10), ampicillin/sulbactam (10/10), ceftriaxone (30), cefuroxime (30), cefepime (30), ciprofloxacin (5), gentamicin (10), levofloxacin (5), tetracycline (30), imipenem (10), meropenem (10), ertapenem (10), trimethoprim (5) and tigecycline (15). Antibiotic susceptibility profiling was also performed using the VITEK-2 system in accordance with the manufacturer’s instructions (BioMerieux^®^, France). Interpretative values obtained by VITEK-2 were compared to those obtained by the disk diffusion method. The following definitions were adopted: (1) Categorical agreement (CA) (VITEK-2 and manual MIC values agree using the interpretative CLSI criteria). (2) Minor errors (mE) (Manual is S or R and VITEK-2 is I; alternatively, Manual is I and VITEK-2 is S or R). (3) Major errors (ME) (Manual is S and VITEK-2 is R). (4) Very major errors (VME) (Manual is R, and VITEK-2 is S). Each assay used *E. coli* ATCC 25,922, *K. pneumoniae* ATCC 700,603, and *A. baumannii* ATCC 19,606 as controls.

### Quantitative biofilm assay

Biofilm formation was carried out according to a previously established protocol^10^, with some modifications. The isolates were cultured overnight in Luria–Bertani Broth (LB) at 37 °C and adjusted to a 0.5 McFarland turbidity standard. One milliliter of the culture was added to 9 mL of LB. From each bacterial suspension, 200 µl was inoculated into three wells of a polyvinyl chloride 96-well microtiter plate and incubated without shaking at 35 °C for 24 h. After incubation, the wells were washed three times with tryptone water and left to air dry for 30 min. Then, 100 µl of 0.5% crystal violet stain was added and left for 15 min. Finally, the wells were washed three times with phosphate-buffered saline, traces of crystal violet were dissolved using 90% ethanol, and the optical density was measured at 600 nm using an ELISA auto reader. The degree of biofilm formation was calculated using the formula; SBF = (AB − CW)/G, where SBF is the specific biofilm formation index, AB is the optical density of the stained bacteria, G is the optical density of cell growth in media and CW is the optical density of the stained control wells containing absolute medium without bacteria. The isolates were classified as follows: SBF ≥ 1.10: strong biofilm formation, SBF = 0.70–1.09: moderate biofilm formation, SBF = 0.35–0.69: weak biofilm formation, and SBF < 0.35: negative biofilm formation.

### Phenotypic detection of ESBL-producing isolates

Isolates were screened for ESBL production using the double disk synergy test (DDST) according to the CLSI 2020^11^. An augmentin disc (30 µg) was placed in the middle of a Mueller-Hinton agar plate seeded with a suspension of an isolate equivalent to 0.5 McFarland turbidity standard. Three antibiotic discs were placed around the augmentin disc: cefepime (30 µg), ceftazidime (30 µg), and ceftriaxone (30 µg). The plate was incubated at 37 °C for 24 h. If the inhibition zone increased toward the augmentin disc, the isolate was considered an ESBL producer.

### PCR conditions

Genomic DNA purification was done using a kit (Thermo Fisher Scientific, United States) according to the manufacturer’s protocol. Three pairs of primers listed in Table 1 were used for the amplification of *16–23 S ribosomal RNA* (*16–23 S rRNA*), *UIDA*, and *OMPA* genes for the identification of *K. pneumoniae*,* E. coli*, and *A. baumannii*, respectively. Each PCR mixture contained 2 µl of each primer (forward and reverse), 10 µl of PCR master mix (Willow Fort, UK), and 4 µl of extracted chromosomal DNA, and 20 µl of each mixture was mixed with sterile nuclease-free water. DNA amplification was performed using a Horizontal Thermocycler (Biometra, Germany). As shown in Table 2, amplification was performed with cycling parameters including initial denaturation at 95˚C for 5 min, followed by 30 cycles each of denaturation at 94˚C for 30 s to 1 min, annealing at 58˚C for 30 s to 1 min, extension at 72˚C, and a final extension at 72˚C for 5 min. The PCR products were analyzed using 2% agarose gel electrophoresis.

**Table 1:** Primer sequence and expected sizes of the PCR product.

Identification Gene	Primer sequence (5′ → 3′)	Expected size (bp)	References
*16–23 S rRNA*	(F) ATTTGAAGAGGTTGCAAACGAT (R) TTCACTCTGAAGTTTTCTTGTGTTC	130	^12^
*UIDA*	(F) TGGTAATTACCGACGAAAACGGC (R) ACGCGTGGTTACAGTCTTGCG	162	^13^
*OMPA*	(F) TCTTGGTGGTCACTTGAAGC (R) ACTCTTGTGGTTGTGGAGCA	85	^14^

F: Forward R: Reverse bp: Base pair.

**Table 2:** PCR thermocycling conditions.

Gene	Initial Denaturation °C/Time	Denaturation °C/Time	Annealing °C/Time	Extension °C/Time	Final extension °C/Time	Number of Cycles	References
*16–23 S rRNA*	94 °C/5 min	95 °C/1 min	58 °C/1 min	72 °C/1 min	72 °C/5 min	30	^12^
*UIDA*	94 °C/3 min	94 °C/30 s	52 °C/30 s	68 °C/30 s	72 °C/5 min	30	^13^
*OMPA*	94 °C/5 min	94 °C/1 min	50°/1 min	72 °C/45 s	72 °C/5 min	30	^14^

### Determination of genetic diversity using ERIC-PCR

Genotyping of the selected *K. pneumoniae* isolates was performed by Enterobacterial Repetitive Intergenic Consensus Polymerase Chain Reaction (ERIC-PCR) using a pair of published primers (F: 50 -ATG TAA GCT CCT GGG GAT TCA C-30) (R: 50 -AAG TAA GTG ACT GGG GTG AGC-30)^15^. The PCR master mix was prepared according to the instructions of Emerald Amp GT PCR master mix (Takara Bio INC, Japan) Code No. RR310A kit. The thermal cycling protocol was performed as follows: initial denaturation of the target DNA sequence at 94 ^◦^C for 5 min, followed by 35 cycles of secondary denaturation at 94 ^◦^C for 30 s, annealing at 52 ^◦^C for 1 min, extension of the primers by thermostable polymerase at 72 ^◦^C for 2 min and a final extension step for 12 min at 72 ^◦^C, followed by cooling to 4 ^◦^C. Electrophoresis of PCR products was done using 1.5% agarose gel. The ERIC fingerprinting data were transformed into a binary code depending on the presence or absence of each band. Dendrograms were generated by the unweighted pair group method with arithmetic average (UPGMA) and Ward’s hierarchical clustering routine. Cluster analysis and dendrogram construction were performed with SPSS version 22 (IBM 2013)^16^. The discriminatory index (D-value) was calculated using an online discriminatory power calculator (http://insilico.ehu.es/mini_tools/discriminatory_power/). The similarity index (Jaccard/Tanimoto Coefficient and number of intersecting elements) between all samples was calculated using an online tool (https://planetcalc.com/1664/)^17^.

### Phenotypic identification of hypervirulent *Klebsiella pneumoniae*

The selected *K. pneumoniae* isolates were cultured on MacConkey agar. The incubation was set at 37 °C for 24 h under aerobic conditions. A string test was carried out by gently stretching the colonies using the bacteriological loop. The test is considered positive, and the strain is if a mucoid string with > 5 mm in length is observed^18^.

### Virulome screening by PCR

Profiling of the virulence genes among *K. pneumoniae*,* E. coli*, and *A. baumannii* was performed, including the *EAST-1*,* CNF-1*,* FimH*,* rmpA*,* iutA*,* FyuA*,* bla*_*SHV−1*_, *bla*_*TEM−1*_, *bla*_*KPC*,_ and *bla*_*NDM*_ genes. All primers used are listed in Table 3. As shown in Table 4, amplification was performed with cycling parameters including initial denaturation at 95˚C for 4–5 min, except for the *bla*_*KPC*,_ and *bla*_*NDM*_ genes were for 15 min, followed by 30 cycles each of denaturation at 94˚C for 30–90 s. Different annealing temperatures were employed depending on the detected gene, ranging from 50–63˚C for 40–90 s. Followed by extension at 72˚C, and a final extension at 72˚C for up to 10 min was employed. The PCR products were analyzed using 2% agarose gel electrophoresis.

**Table 3:** Primer sequences and expected sizes of the PCR products.

Virulence genes	Primer sequence (5′ → 3′)	Expected size (bp)	References
*EAST-1*	(F) ATGCCATCAACACAGTATAT (R) GCGAGTGACGGCTTTGTAGT	110	^19^
*CNF-1*	(F)AAGATGGAGTTTCCTATGCAGGAG (R)CATTCAGAGTCCTGCCCTCATTATT	498	^19^
*FimH*	(F) TGCAGAACGGATAAGCCGTGG (R) GCAGTCACCTGCCCTCCGGTA	508	^20^
*rmpA*	(F) ACTGGGCTACCTCTGCTTCA (R) CTTGCATGAGCCATCTTTCA	535	^12^
*iutA*	(F) GGCTGGACATCATGGGAACTGG (R) CGTCGGGAACGGGTAGAATCG	300	^21^
*FyuA*	(F) TGATTAACCCCGCGACGGGAA (R) CGCAGTAGGCACGATGTTGTA	880	^20^
*bla*_*SHV−1*_	(F) GGCCGCGTAGGCATGATAGA (R) CCCGGCGATTTGCTGATTTC	714	^22^
*bla*_*TEM−1*_	(F) CAGCGGTAAGATCCTTGAGA (R) ACTCCCCGTCGTGTAGATAA	643	^22^
*bla*_*KPC*_	(F) CGTCTAGTTCTGCTGTCTTG (R) CTTGTCATCCTTGTTAGGCG	798	^23^
*bla*_*NDM*_	(F) GGTTTGGCGATCTGGTTTTC (R) CGGAATGGCTCATCACGATC	621	^23^

F: Forward R: Reverse bp: Base pair.

**Table 4:** PCR thermocycling conditions.

Gene	Initial Denaturation °C/Time	Denaturation °C/Time	Annealing °C/Time	Extension °C/Time	Final Extension °C/Time	Number of Cycles	References
*EAST-1*	95 °C/4 min	94 °C/50s	58 °C/1.5 min	72 °C/1.5 min	72 °C/10 min	35	^24^
*CNF-1*	95 °C/4 min	94 °C/50s	58 °C/1 min	72 °C/45s	72 °C/8 min	30	^20^
*FimH*	95 °C/4 min	94 °C/1 min	56 °C/45s	72 °C/1 min	72 °C/10 min	34	^20^
*rmpA*	94 °C/4 min	94 °C/30s	50 °C/40s	72 °C/1 min	72 °C/10 min	30	^22^
*iutA*	94 °C/4 min	94 °C/30s	63 °C/40s	72 °C/1 min	72 °C/10 min	30	^21^
*FyuA*	95 °C/4 min	95 °C/50s	58 °C/1 min	72 °C/45s	72 °C/8 min	30	^20^
*bla*_*SHV−1*_	95 °C/5 min	94 °C/30s	55 °C/1 min	72 °C/45s	72 °C/7 min	30	^22^
*bla*_*TEM−1*_	95 °C/5 min	94 °C/30s	52 °C/45s	72 °C/45s	72 °C/7 min	30	^22^
*bla*_*KPC*_	95 °C/15 min	94 °C/30s	58 °C/90s	72 °C/90s	72 °C/10 min	35	^23^
*bla*_*NDM*_	95 °C/15 min	94 °C/30s	58 °C/90s	72 °C/90s	72 °C/10 min	35	^23^

### Statistical analysis

Statistical analysis was performed with IBM Statistical Package for Social Sciences (SPSS) Statistics for Windows Version 23.0. Armonk, NY: IBM Corp. Fisher’s exact test was used to assess the correlation between MDR isolates and biofilm formation. Pearson’s chi-squared test was used to compare between the biofilm producing capacity of different isolates and analyze the difference between the DDST and VITEK-2 systems in the detection of ESBL producers. A probability value (*P* value) less than 0.05 was considered to indicate statistical significance.

## Results

### Microbial population

Among the 231 blood specimens, 185 were positive. Of these, 185 microbial isolates were recovered. Laboratory examination of the isolates revealed that 120 (64.4%), 56 (30.2%), and 9 (4.8%) were gram-negative, gram-positive, and *Candida* spp., respectively. Among the gram-negative isolates, the most common organisms identified were *K. pneumoniae* (51; 27.5%), followed by *E. coli* (46; 24.8%), *A. baumannii* (11; 5.9%), *P. aeruginosa* (7; 3.7%), *Salmonella* (3; 1.62%), and *S. marcescens* (2; 1%).

### Antimicrobial susceptibility testing

The antimicrobial susceptibility profiles of *K. pneumoniae*, *E. coli*, *A. baumannii*, and *P. aeruginosa* were determined using the disk diffusion method and the VITEK-2 system. Among the 120 recovered GNB strains, 76.6% (92/120) were MDR-GNB strains as they were resistant to more than three antibiotic classes, 57.5% (69/120) were CRE as they exhibited intermediate or resistance designations to more than one of the tested carbapenems. A comparison between the results of the VITEK-2 system and the disk diffusion method for *K. pneumoniae* and *E. coli* is presented in (S1). For the *K. pneumoniae* isolates, CA was 100% for cefepime, tetracycline, trimethoprim, and tigecycline; 98% for levofloxacin and gentamicin; 96% for amikacin, imipenem, and ciprofloxacin; and 94.1% for ampicillin, ceftriaxone, and cefuroxime.

For the *E. coli* isolates, CA was 100% for amikacin, trimethoprim, and tigecycline; 97.8% for imipenem, gentamicin, and levofloxacin; 95.6% for cefuroxime, cefepime, and tetracycline; and 93.4% for ampicillin, ampicillin + sulbactam, ertapenem, meropenem, and ciprofloxacin. A categorical agreement of 100% was found with the tested antibiotics for the *A. baumannii* and *P. aeruginosa* isolates.

### Quantitative biofilm assay

As shown in Table 5, among the *K. pneumoniae* strains, 47% were strong biofilm producers, 31.3% were moderate producers, 9.8% were weak producers, and 11.7% were non-biofilm producers. The differences between biofilm production within *K. pneumoniae* strains were statistically significant (*P*-value < 0.001). For *E. coli*, 50% were strong biofilm producers, 30.4% were moderate, 9.8% were weak, and 8.6% were non-biofilm producers. The differences between biofilm production within *E. coli* strains were statistically significant (*P*-value < 0.001). For the *A. baumannii* isolates, 63.6% were strong biofilm producers, and 36.3% were moderate, with a non-statistically significant difference (*P*-value = 0.366). For *P. aeruginosa*, 42.85% were strong biofilm producers, 28.5% were moderate producers, and 28.5% were non-biofilm producers. The differences between biofilm production within *P. aeruginosa* strains were non-statistically significant (*P*-value = 0.368).

Comparison between strong biofilm-producing strains showed a statistically significant difference (*P*-value < 0.001). *K. pneumoniae* strains showed the highest prevalence among strong biofilm-producing strains, followed by *E. coli*, and then *A. baumannii* strains, while *P. aeruginosa* strains showed the lowest production of strong biofilm. Comparison between moderate biofilm-producing strains also showed a statistically significant difference (*P*-value = 0.005). *K. pneumoniae* strains showed the highest prevalence among moderate biofilm-producing strains, followed by *E. coli*, while *A. baumannii* and *P. aeruginosa* strains showed the same and the lowest production of moderate biofilm. While for weak and non-biofilm production, there was no statistically significant difference between strains (*P*-value = 1) and (*P*-value = 0.368), respectively. Overall, *K. pneumoniae* was consistently the most prevalent strain in both strong and moderate biofilm categories.

**Table 5:** Biofilm-producing capacity of different Gram-negative isolates.

Microorganism	Strong Biofilm producer *n* (%)	Moderate Biofilm producer *n* (%)	Weak Biofilm producer *n* (%)	Non-Biofilm producer *n* (%)	*P*-value
*K. pneumoniae* (*n* = 51)	24 (47%)	16 (31.3%)	5 (9.8%)	6 (11.7%)	< 0.001*
*E. coli* (*n* = 46)	23 (50%)	14 (30.4%)	5 (9.8%)	4 (8.6%)	< 0.001*
A. *Baumannii* (*n* = 11)	7 (63.6%)	4 (36.3%)	**-**	**-**	0.366
*P. aeruginosa* (*n* = 7)	6 (42.8%)	4 (28.5%)	**-**	2 (28.5%)	0.368
*P*-value	< 0.001*	0.005*	1	0.368	

*: Significant at *P* ≤ 0.05, n: number.

### Association of biofilm formation and antibiotic resistance

Among the biofilm producers, GNB, 68.6% of the *K. pneumoniae* isolates were MDR (*P* value = 0.016), and 84.7% of the *E. coli* isolates were MDR (*P* value = 0.001). Thus, there was a significant correlation between MDR phenomena and biofilm formation among *K. pneumoniae* and *E. coli* isolates.

### Phenotypic detection of ESBL-producing isolates

Using DDST, 50.9% of *K. pneumoniae* and 67.3% of *E. coli* strains were ESBL producers, while the results of the VITEK-2 system revealed that 56.8% of *K. pneumoniae* and 71.7% of *E. coli strains* were ESBL producers. Accordingly, no statistically significant difference was found between the two methods for *K. pneumoniae* and *E. coli*, with *P* values of 0.355 and 0.205, respectively.

### Identification of selected *K. pneumoniae* isolates using PCR

PCR analysis of the ten isolates of *K. pneumoniae*,* E. coli*, and *A. baumannii* revealed the presence of the *16–23 S rRNA*,* UIDA*, and *OMPA* genes, respectively, thus confirming their identification.

### ERIC-PCR analysis

**Fig. 1:**
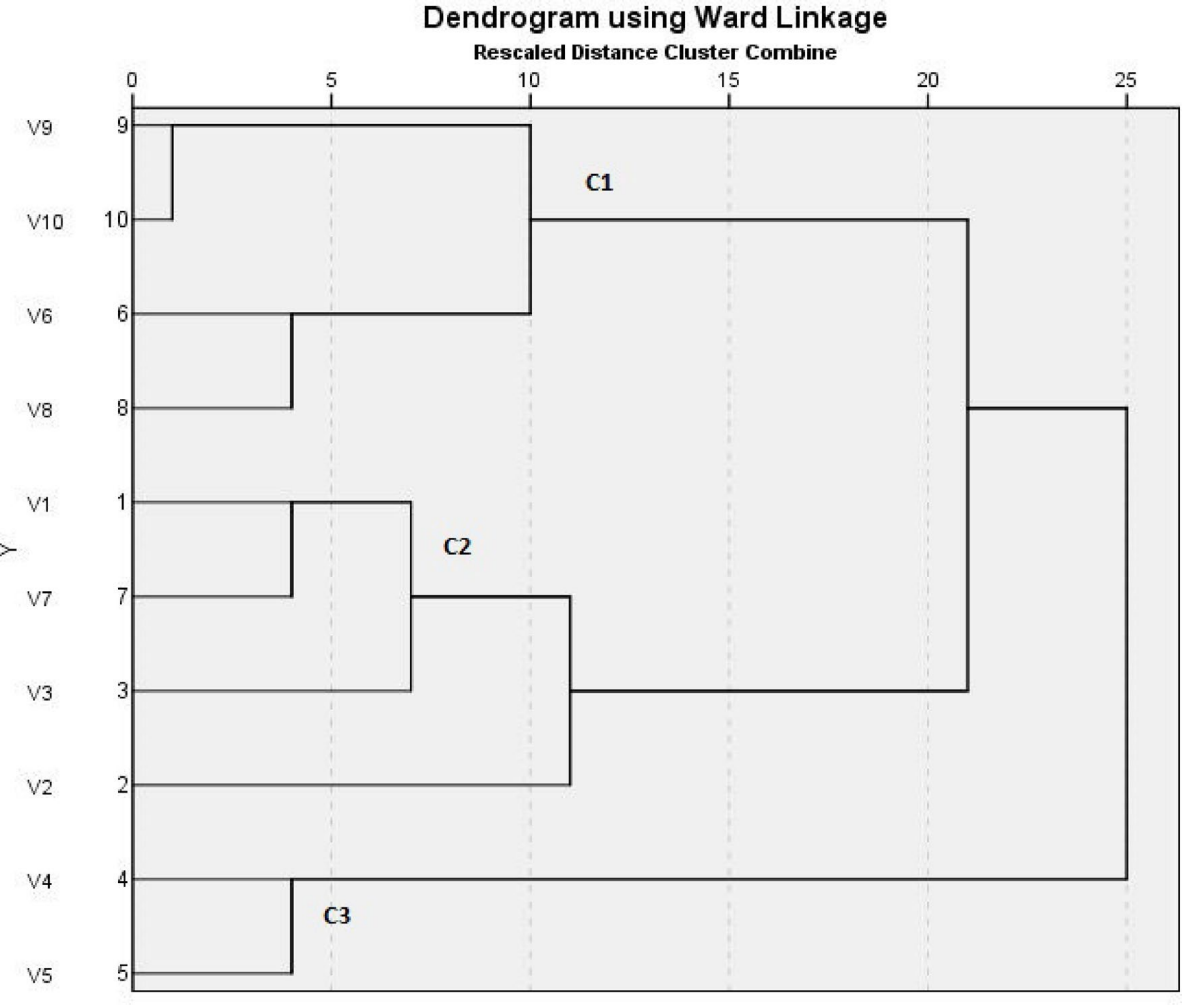
Dendrogram of 10 *K. pneumoniae* isolates showing genetic heterogeneities.

The variable banding pattern of ERIC-PCR gel electrophoresis revealed diversity among the 10 selected *K. pneumoniae* isolates. The DNA bands yielded from REP-PCR type amplification were thoroughly analyzed, and a phylogenetic tree for the isolated strains was designed by GelClust. The cluster analysis and related dendrogram are shown in Fig. 1. Based on the results shown in Fig. 1, the *K. pneumoniae* isolates were categorized into three clusters (C1–C3) with a discriminatory power of 0.7111, which is closer to 1.0 than 0, revealing wide heterogeneities among the tested isolates. Dendrogram analysis revealed an overall similarity of 73.3% among the three clusters. Furthermore, the similarity between each C1–C3 cluster member was 87.5%, 75.5%, and 57%, respectively.

### String test

The ten selected *K. pneumoniae* isolates were positive for the string test as the string length was ≥ 0.5 mm

### Prevalence of virulence and resistance genes among *K. pneumoniae*

**Fig. 2:**
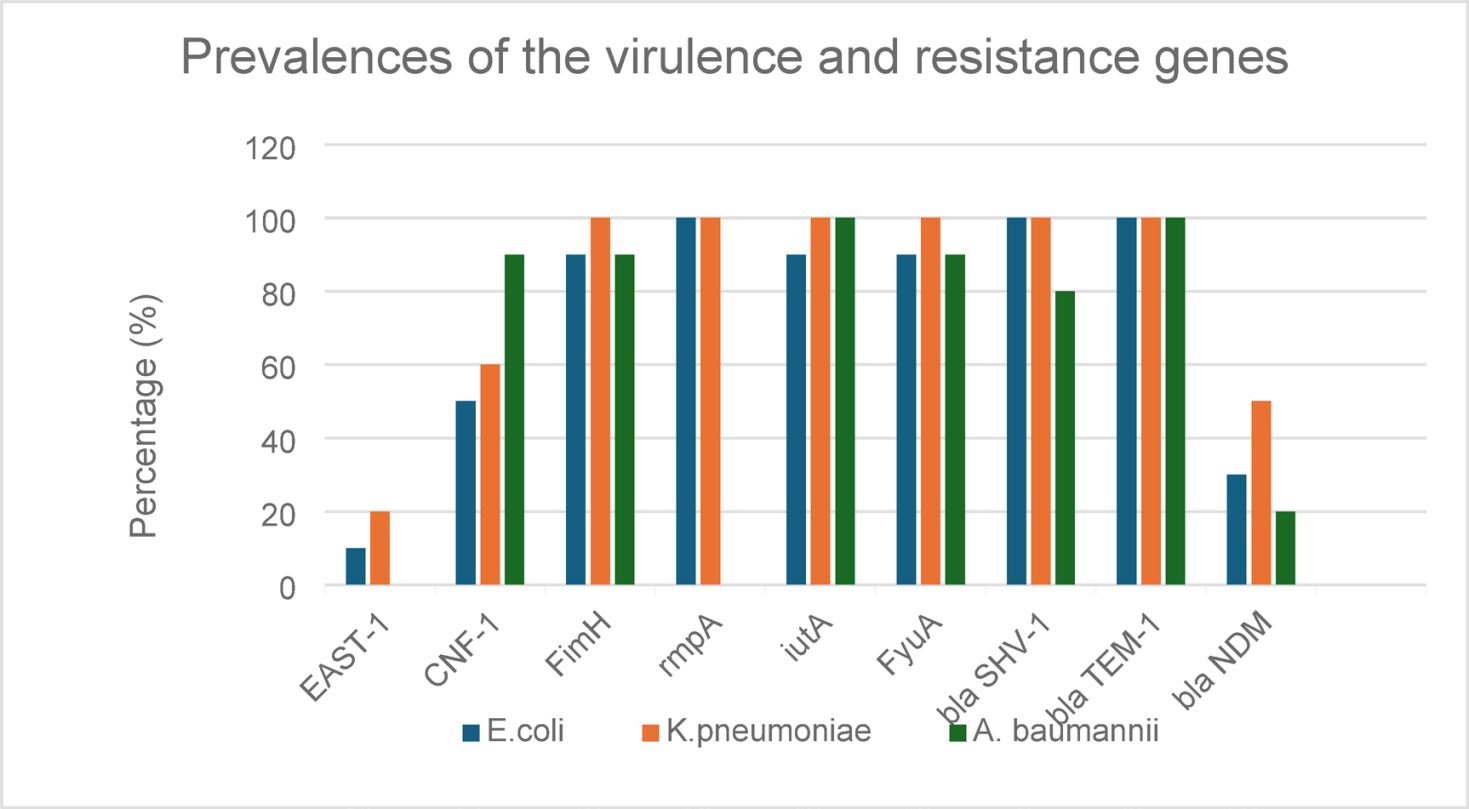
Detecting the prevalence of virulence and resistance genes among *Klebsiella pneumoniae*,* Escherichia coli*, and *Acinetobacter baumannii* isolates.

As shown in Fig. 2, the genes responsible for toxin production in *K. pneumoniae* (*EAST-1* and *CNF-1*) were present in 20% and 60% of the selected isolates, respectively. All isolates contained genes associated with biofilm formation (*FimH*), the iron acquisition gene (*iutA*), and the gene linked to hypermucoviscosity (*rmpA*). The other iron acquisition gene (*FyuA*) was found in 90% of the isolates. Additionally, all isolates harbored the two ESBL genes, bla_*TEM−1*_ and bla_*SHV−1*_, and the carbapenemase resistance gene bla_*NDM*_ was found in 50% of the isolates. The other carbapenemase resistance gene, bla_*KPC*_, was not detected in any of the isolates. In comparison, *E. coli* and *A. baumannii* exhibited a lower prevalence of virulence genes than *K. pneumoniae*. Regarding resistance genes, both ESBL genes were present in all *E. coli* isolates, while the bla_*TEM−1*_ gene was found in 80% of A. baumannii isolates. The prevalence of bla_*NDM*_ was 30% in *E. coli* and 20% in *A. baumannii*.

### Correlation between the string test and RmpA and ItuA genes

All isolates were positive for the string test and harbored the *rmpA* and *ituA* genes, thus confirming their identification as hvPk.

### Correlation between biofilm formation, ESBL production, and their related genes

All isolates were found to be both biofilm-forming and ESBL-producing and harbored their related genes, the *FimH* gene (for biofilm formation) and the *bla*_*TEM−1*_ and *bla*_*SHV−1*_ genes (for ESBL production).

## Discussion

Globally, bloodstream infections (BSIs) are among the leading and most life-threatening conditions in hospital settings, particularly in immunocompromised patients^25^. Monitoring antibiotic susceptibility patterns and virulence genes in pathogens causing BSIs is crucial for determining the optimum antimicrobial therapy for patients^26^. Among the Gram-negative pathogens, *K. pneumoniae* is one of the frequent causes of BSIs associated with increased mortality and commonly occurring in patients with severe underlying conditions such as malignancies and trauma^27,28^. Therefore, this study focused on virulome analysis and antibiotic resistance pattern of *K. pneumoniae* in comparison with *E. coli* and *A. baumannii*, with emphasis on hypervirulent strains of *K. pneumoniae*.

Our results revealed the predominance of gram-negative bacteria (64.8%), with *K. pneumoniae* followed by *E. coli* being the most isolated pathogens from BSIs, with percentages of 27.5% and 24.8%, respectively. Similar findings were previously reported in other countries^29,30^. Such findings could be attributed to being in the hospital for more than one week with a catheter inserted for more than 3 days, undergoing surgery, or receiving treatment with antimicrobial agents such as beta-lactams^31^. Moreover, our results were in accordance with other studies conducted in Egypt, revealing the predominance of *K. pneumoniae* among BSIs over the past few decades^32,33^.

Biofilm formation is an important virulence factor in Gram-negative pathogens, significantly enhancing resistance to external stressors. This mechanism allows pathogens to evade host immune responses and the antibacterial effects of antibiotics, contributing to the challenges in treating various diseases and potentially leading to therapeutic failures. Biofilm formation can also result in the emergence of MDR strains, which are linked to poorer clinical outcomes and increased mortality rates, especially among immunocompromised patients^34,35^. The results of the quantitative biofilm assay revealed that among GNB, 88.3% of *K. pneumoniae*, 91.4% of *E. coli*, 100% of *A. baumannii*, and 71.5% of *P. aeruginosa* were biofilm producers. Previous literature reported that around 84% of Gram-negative bacteria isolated from CVC-related infections were biofilm producers^36^.

In this study, the overall proportion of MDR-GNB was 76.6% (92/120). The significant multidrug-resistant (MDR) patterns observed in various Gram-negative pathogens pose a major challenge in managing infectious diseases. Therefore, optimizing antibiotic use through stewardship programs is essential, as these initiatives are key components in the battle against antimicrobial resistance^37^. Numerous studies indicate that using a combination of antibiotics can help prevent the development of new resistant strains, as treatment failures are often seen in patients receiving only single antibiotic therapy. Additionally, collaboration between clinicians and microbiologists is crucial for effective infection management, as emphasized by the Rational Use of Medicine Program. *K. pneumoniae* is one of the most important causes of MDR infections worldwide. This high prevalence is not surprising, as this pathogen is known for its ability to transfer its resistance determinants and its association with BSIs^38^. This study proved that a significant correlation was found between MDR phenomena and biofilm formation among *K. pneumoniae* and *E. coli* isolates, with *P* values of 0.016 and 0.001, respectively. The correlation between biofilm formation and antibiotic resistance is of great concern, especially in healthcare facilities for patients with device-related infections. These results were in line with a previous study conducted by Nirwati et al. (2019), where antibiotic resistance was greater among biofilm producers of *K. pneumoniae* than non-biofilm producers^38^.

The widespread occurrence of MDR isolates, especially ESBL producers, has presented a global threat to public health^39^. In our study, the prevalence of ESBL production was detected by DDST and VITEK-2, revealing a high prevalence among *K. pneumoniae* and *E. coli*, with no significant difference between the two methods. Such a high prevalence could be attributed to the empirical use of antibiotics, resulting in positive pressure on GNB, leading to the selection of resistant strains^40^.

ERIC-PCR analysis demonstrated a discriminatory power of 0.7111 among the selected isolates, confirming significant heterogeneity among the tested strains. Consequently, these isolates were assessed for hypervirulence, as hvKp poses a growing threat as a pathogen responsible for life-threatening infections and is crucial to differentiate from classical *K. pneumoniae*^41^. As previously mentioned by Emam et al. (2023), hvKp can be identified phenotypically using the string test. All our isolates were found to be hvKp since hypermucoviscosity is one of the typical features of hvKp^42^.

Several previous studies from different countries focused on evaluating the prevalence of important virulence genes in *K. pneumoniae*^43,44^. Consequently, the study focused on investigating the prevalence of selected crucial virulence genes encoding for biofilm formation (*FimH*), the gene associated with hypermucoviscosity (*rmpA*), the iron acquisition genes (*iutA*) and (*fyuA*), as well as the genes encoding toxin production (*EAST-1*,* CNF-1*). According to Anis et al. (2021), the *FimH* gene is strongly linked to biofilm formation^45^. We detected this gene and confirmed that all *K. pneumoniae* possessed the *FimH* gene, indicating they are biofilm producers. A slightly lower prevalence of this gene was found in *E*. *coli* and *A. baumannii.* Our results revealed that *K. pneumoniae* strains showed the highest prevalence among strong and moderate biofilm-producing strains, with a statistically significant difference. Moreover, research shows that when comparing the biofilm-forming abilities of *E. coli* and *K. pneumoniae*, *Klebsiella* is generally more effective at forming biofilms, especially in clinical settings, which plays a significant role in its virulence and resistance to treatment^46^.

The *rmpA* gene is a mucoid regulator that mediates the increased capsular polysaccharide production and is one of the genotypic markers for identifying hvKp. This direct correlation has been mentioned before by Neumann et al. (20230) and by Khattab and Hager (2022)^47,48^. Our results confirmed the presence of the *rmpA* gene in all selected isolates, which is considered one of the key virulent features of *K. pneumoniae*. Although this gene has been found in all *E. coli* isolates, the *rmpA* gene is not considered part of the core genome of *E. coli*. However, its presence contributes to the capsule formation and can enhance the ability of bacteria to resist certain host defenses^49^. As previously reported by Shankar et al. (2021), the aerobactin (*iutA*) gene is another frequent and stable genotypic marker for hvKp isolates; our investigations also detected the (*iutA*) gene and confirmed its presence in all selected isolates^50^. Together, these genes significantly enhance the hypervirulent phenotype of *K. pneumoniae*, particularly in central line-associated bloodstream infections (CLABSIs). Increased biofilm formation, effective iron acquisition, and strong adherence capabilities allow *K. pneumoniae* to establish and maintain infections, leading to severe clinical outcomes^46^.

As iron is one of the essentials for bacterial survival and reproduction, we also assessed the presence of the yersiniabactin receptor gene (*fyuA*), another important iron uptake gene besides the *iutA* gene^51^. The *FyuA* gene has been identified in 90% of *K. pneumoniae* isolates, although several studies have reported finding it at lower frequencies^52^. Iron is essential for the survival, growth, and pathogenicity of *A. baumannii*. To thrive in the iron-limited environment of the host, this bacterium has developed multiple iron acquisition systems, including the production of siderophores, heme uptake mechanisms, and TonB-dependent transport systems^53^. This clarifies the reason for the high prevalence of the *FyuA* and the *iutA* genes in *A. baumannii*. Moreover, the iron acquisition genes are a crucial factor contributing to the pathogenicity of *E. coli*, accordingly, a high prevalence of these genes was found among the *E. coli* isolates, which was also previously reported by Guo et al. (2024)^54^.

The prevalence of virulence genes responsible for toxin production, such as the *EAST-1* and *CNF-1* genes, has been assessed. Although no previous study detected the *EAST-1* gene among *K. pneumoniae*, our results confirmed its low prevalence. Moreover, a DNA search in the GenBank database carried out using BLAST (Basal Local Alignment Research Tool) records the presence of the gene’s sequence in several *K. pneumoniae* strains (https://www.ncbi.nlm.nih.gov/nucleotide/1890420180). The *CNF-1* gene was detected in 60% of our *K. pneumoniae* isolates. Similarly, Lateef et al. (2012) identified the *CNF-1* gene in 57.1% of *K. pneumoniae* isolates^55^. In the case of *E. coli*, 50% of the isolates carried the *CNF-1* gene, whereas 90% of *A. baumannii* isolates possessed it. A study conducted by Sheikh et al. (2019) found the *CNF-1* gene in 22.8% of *E. coli* isolates^56^. As for *A. baumannii* isolates, it was reported by Al-Kadmy et al. (2018) that the *CNF-1* gene was detected in 47.6% of their isolates^57^. The high prevalence of the *CNF-1* gene underscores the importance of this gene in the virulence of *A. baumannii* strains.

The detection of the resistance determinants *bla*_*SHV−1*_ and *bla*_*TEM*_ genes in all *K. pneumoniae* and *E. coli* isolates confirmed them as ESBL producers. Moreover, both conventional antimicrobial susceptibility testing and the VITEK-2 system verified these isolates as carbapenem-resistant. However, detection of the carbapenemase resistance genes bla_*NDM*_ and bla_*KPC*_, showed that none of the isolates harbored the bla_*KPC*_, while the prevalence of the bla_*NDM*_ among *K. pneumoniae*,* E. coli*, and *A. baumannii* was 50%,30%, and 20%, respectively.

The alarming prevalence of beta-lactamase enzymes, along with the global emergence of carbapenemase enzymes in *K. pneumoniae* strains, is a major public concern due to limited treatment options and emerging resistance to last-resort antibiotics like colistin and tigecycline. Genomic studies show the clonal expansion of high-risk strains, such as ST11, contributing to healthcare outbreaks. The combination of hypervirulence and multidrug resistance poses an even greater threat. While advances in genomic surveillance, phage therapy, and new antibiotics like cefiderocol provide hope, addressing MDR *K. pneumoniae* requires antimicrobial stewardship and global collaboration^58,59^. By implementing effective infection control measures, screening for the microbial source, optimizing antimicrobial use, and discovering novel therapeutic options, it is possible to mitigate these challenges and improve patient outcomes.

### Limitations

This study has several limitations that must be acknowledged. The sample size is relatively small, and the research was conducted at a single center, which may impact the generalizability of the findings. Additionally, the lack of clinical correlation with the study results is another limitation. These challenges arose from logistical issues related to the collection of blood specimens from hospitals and the follow-up of patients. Future studies should aim for larger sample sizes and multi-center involvement to improve the robustness and generalizability of the findings.

## Conclusion

*K. pneumoniae* is a frequent and critical cause of BSIs. As shown in our study, all selected isolates were found to be both ESBL producers and CRE, emphasizing the high level of multidrug resistance encountered nowadays. In comparison to *E. coli* and *A. baumannii*,* K. pneumoniae* was found to be the most persistent biofilm producer. The findings of this study also revealed a significant correlation between biofilm formation and multidrug resistance. Also, a direct correlation has been found between the phenotypic and genotypic detection of various virulence factors, including biofilm formation, hypermucoviscosity, and ESBL production.

Bloodstream infections caused by hyKp strains pose an increasing public health threat in healthcare settings. Consequently, the data collected from our study will contribute to addressing this challenge and identifying better therapeutic alternatives for patient treatment, while also aiming to reduce complications, mortality, and financial burdens.

## Electronic supplementary material

Below is the link to the electronic supplementary material.

Supplementary Material 1

## Data Availability

The datasets generated and/or analysed during the current study are not publicly available due to confidentiality and privacy concerns but are available from the corresponding author upon reasonable request.
