# Supplementary material for: Exploring virulence factors, virulome, and multidrug resistance of Klebsiella pneumoniae strains isolated from patients with central Line-associated bloodstream infections
Source: Sci Rep. 2025 Jun 20;15:20230. doi: 10.1038/s41598-025-07493-6 (PMC12181432; doi:10.1038/s41598-025-07493-6)
Supplement: Supplementary file 1 — Supplementary Material 1 [file 41598_2025_7493_MOESM1_ESM.docx]

**S1 Comparison between the susceptibility pattern of *K. pneumoniae* and *E. coli* using VITEK-2 and disk diffusion method.**

| **Antimicrobial agent** | *K. pneumoniae* | | | | | | | | *E. coli* | | | | | | | | |
| --- | --- | --- | --- | --- | --- | --- | --- | --- | --- | --- | --- | --- | --- | --- | --- | --- | --- |
|  | **CA** | | **mE** | | **ME** | | **VME** | | **CA** | | **mE** | | **ME** | | **VME** | | |
|  | No. | % | No. | % | No. | % | No. | % | No. | % | No. | % | No. | % | No. | % |  |
| Ampicillin | 48 | 94.1 | 2 | 3.9 | 1 | 1.9 | 0 | 0 | 43 | 93.4 | 3 | 6.5 | 0 | 0 | 0 | 0 |  |
| Ampicillin+  Sulbactam | 47 | 92.1 | 2 | 3.9 | 2 | 3.9 | 0 | 0 | 43 | 93.4 | 1 | 2.1 | 2 | 4.3 | 0 | 0 |  |
| Ceftriaxone | 48 | 94.1 | 1 | 1.9 | 2 | 3.9 | 0 | 0 | 41 | 89.1 | 1 | 2.1 | 2 | 4.3 | 2 | 4.3 |  |
| Cefuroxime | 48 | 94.1 | 3 | 5.8 | 0 | 0 | 0 | 0 | 44 | 95.6 | 1 | 2.1 | 1 | 2.1 | 0 | 0 |  |
| Cefepime | 51 | 100 | 0 | 0 | 0 | 0 | 0 | 0 | 44 | 95.6 | 0 | 0 | 2 | 4.3 | 0 | 0 |  |
| Ertapenem | 53 | 84.3 | 7 | 13.7 | 0 | 0 | 35 | 36 | 43 | 93.4 | 2 | 4.3 | 1 | 2.1 | 0 | 0 |  |
| Imipenem | 49 | 96 | 0 | 0 | 1 | 1.9 | 36 | 37.1 | 45 | 97.8 | 1 | 2.1 | 0 | 0 | 0 | 0 |  |
| Meropenem | 46 | 90.1 | 3 | 5.8 | 0 | 0 | 36 | 37.1 | 43 | 93.4 | 1 | 2.1 | 2 | 4.3 | 0 | 0 |  |
| Gentamicin | 50 | 98 | 1 | 1.9 | 0 | 0 | 24 | 24.7 | 45 | 97.8 | 0 | 0 | 1 | 2.1 | 0 | 0 |  |
| Amikacin | 49 | 96 | 0 | 0 | 1 | 1.9 | 48 | 49.4 | 46 | 100 | 0 | 0 | 0 | 0 | 0 | 0 |  |
| Tetracycline | 51 | 100 | 0 | 0 | 0 | 0 | 16 | 16.4 | 44 | 95.6 | 1 | 2.1 | 1 | 2.1 | 0 | 0 |  |
| Ciprofloxacin | 49 | 96 | 0 | 0 | 2 | 3.9 | 18 | 18.5 | 43 | 93.4 | 1 | 2.1 | 1 | 2.1 | 1 | 2.1 |  |
| Levofloxacin | 50 | 98 | 1 | 1.9 | 0 | 0 | 19 | 19.5 | 45 | 97.8 | 1 | 2.1 | 0 | 0 | 0 | 0 |  |
| Trimethoprim | 51 | 100 | 0 | 0 | 0 | 0 | 16 | 16.4 | 46 | 100 | 0 | 0 | 0 | 0 | 0 | 0 |  |
| Tigecycline | 51 | 100 | 0 | 0 | 0 | 0 | 90 | 92.7 | 46 | 100 | 0 | 0 | 0 | 0 | 0 | 0 |  |

**VME:** Very major error, **ME:** Major error**, mE:** Minor error**, CA:** Categorical agreement
